# Surgical resection of extremely rare primary giant splenic angiomyolipoma: a case report

**DOI:** 10.1186/s40792-021-01192-w

**Published:** 2021-05-03

**Authors:** Kaoru Sato, Fumito Saijo, Yu Katayose, Mitsuhisa Mutoh, Noriyuki Iwama, Fumie Nakayama, Hiromi Tokumura

**Affiliations:** 1 Department of Surgery, Tohoku Rosai Hospital, 4-3-21, Dainohara, Aobaku, Sendai, Miyagi 981-8563 Japan; 2 Department of Surgery, Tohoku University Hospital, 1-1, Seiryo-machi, Aobaku, Sendai, Miyagi 980-8574 Japan; 3 Division of Gastroenterologic and Hepato-Biliary-Pancreatic Surgery, Department of Surgery, Tohoku Medical and Pharmaceutical University, 1-12-1 Fukumuro, Miyagino-ku, Sendai, 983-8512 Japan; 4 Department of Pathology, Tohoku Rosai Hospital, 4-3-21, Dainohara, Aobaku, Sendai, Miyagi 981-8563 Japan

**Keywords:** Spleen, Giant tumor, Primary angiomyolipoma

## Abstract

**Background:**

Angiomyolipoma is a benign mesenchymal tumor that develops commonly in the kidney and rarely in other organs. The involvement of the spleen in angiomyolipoma is extremely rare, and only one such case has been reported in the English literature.

**Case presentation:**

A 27-year-old man presented with adenoid hyperplasia and bilateral palatal tonsillar hyperplasia. During the treatment for adenoid hyperplasia, a 15-cm tumor was detected in the spleen using abdominal ultrasonography and enhanced computed tomography. Partial resection of the spleen was successfully performed. A giant tumor of approximately 13 cm with a smooth surface was observed in the upper left quadrant of the abdomen. The tumor was confirmed to be continuous with the upper spleen, and there was no invasion of the other organs. The postoperative course was good, and the patient was discharged on the 7th postoperative day. The excised specimen was a smooth, extremely soft tumor measuring 123 × 120 × 82 mm. The cleaved surface of the tumor was reddish brown, and a distressing yellow color was observed. Pathological examination revealed a proliferation of mature adipocytes and an increase in the number of blood vessels of various sizes. Furthermore, spindle-shaped cell proliferation foci were visible between the adipocytes and the surrounding blood vessels. Profuse leakage of erythrocytes from the blood vessels, hemosiderin deposition, and small round cell infiltration were also noted. Immunostaining disclosed that the spindle-shaped cells were weakly positive for smooth muscle antibody and were identified as smooth muscle cells. The adipocytes and spindle cells were negative for HMB 45, Melan A, MDM, and CDK4. However, some parts of the cells were positive for estrogen and progesterone receptors. Besides, vascular endothelial cells were positive for CD31 and CD34 and negative for CD8. Based on these findings, the patient was diagnosed to have primary angiomyolipoma of the spleen.

**Conclusions:**

We have reported the surgical treatment for an extremely rare case of giant splenic angiomyolipoma in a young man. Globally, this is the second report on this condition. We believe that partial splenic resection is a feasible option for the management of giant tumors.

## Background

Angiomyolipoma is a benign mixed mesenchymal tumor consisting of three elements, namely the blood vessels, smooth muscle cells, and adipocytes. These tumors often develop in the kidney and rarely in other organs such as the liver and uterus [1]. However, primary angiomyolipoma of the spleen is extremely rare. Here, we report an extremely rare case of primary angiomyolipoma of the spleen, which was treated with partial splenic resection.

## Case presentation

A 27-year-old man presented with adenoid hyperplasia and bilateral palatal tonsillar hyperplasia. Bilateral tonsillectomy was performed for adenoid hyperplasia. Abdominal ultrasonography (AUS) and enhanced computed tomography (CT) performed as part of the preoperative examination revealed the presence of a 15-cm tumor in the spleen. The patient was obese (height, 164.8 cm; weight, 128.8 kg; body mass index, 47.4 kg/m^2^) and did not have tuberous sclerosis (TS). His laboratory findings were within the normal range (white blood cell count, 8800/µL; red blood cell count, 433 × 10^4^/µL; hemoglobin, 12.7 g/dL; platelet count, 17.4 × 10^4^/µL; glycated hemoglobin, 6.3%; and C-reactive protein, 0.32 mg/dL).

**Fig. 1:**
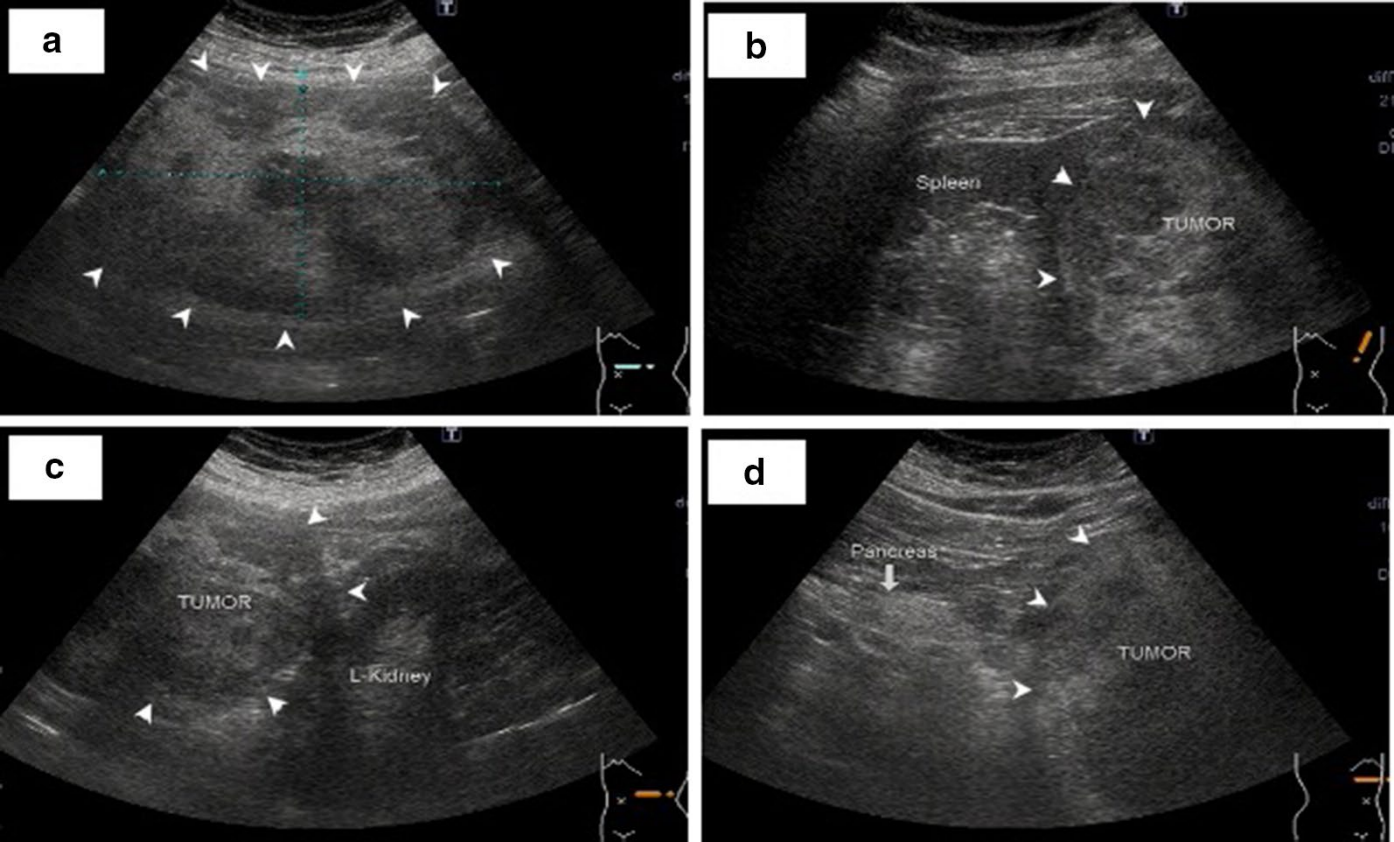
Abdominal ultrasonography. Arrows indicate the tumor. **a** A solid tumor measuring 140 × 128 × 99 cm in the upper left abdomen with uneven hyperechoic and hypoechoic areas. **b** The tumor was continuous with the upper pole of the spleen. **c**, **d** Although it was in contact with the left kidney and pancreas, it was discontinuous

**Fig. 2:**
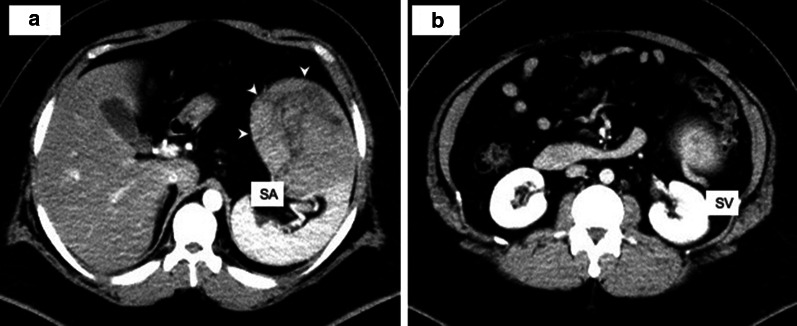
Enhanced computed tomography. Enhanced computed tomography showing a solid tumor (arrow) on the ventral side of the spleen with a clear border of approximately 14 cm continuous with the spleen. The inside mainly contained fat components around the blood vessels, while the solid part was imaged incrementally (**a**). Blood was supplied from the splenic artery (**a**), and dilated blood vessels, thought to be drainage veins, were found in the tumor to the caudal side, joining the splenic vein (**b**). *SA* splenic artery, *SV* splenic vein

AUS showed a solid tumor of approximately 14 cm in the spleen, and the tumor was found to be in contact with the pancreatic tail, left kidney, and stomach. The edges were smooth; the inside was uneven with mixed high- and low-echoic masses (Fig. 1). Enhanced CT revealed a solid tumor of approximately 14 cm with a clear border on the ventral side of the spleen, which was continuous with the spleen. The inside mainly contained fat components around the blood vessels, and the solid part was imaged incrementally. The splenic artery supplied blood to the tumor. A dilated blood vessel, thought to be a vein draining into the splenic vein, was found on the caudal side of the tumor. Only hemangioma was seen in the liver, and no other findings were noted in the pancreas, liver, or kidney (Fig. 2). Magnetic resonance imaging (MRI) could not be performed because of the patient’s claustrophobia.

The tumor was primarily located in the spleen and could not be adequately diagnosed. However, it was large, and the possibilities of rupture and malignancy could not be ruled out. Hence, we opted for a surgical resection.

**Fig. 3:**
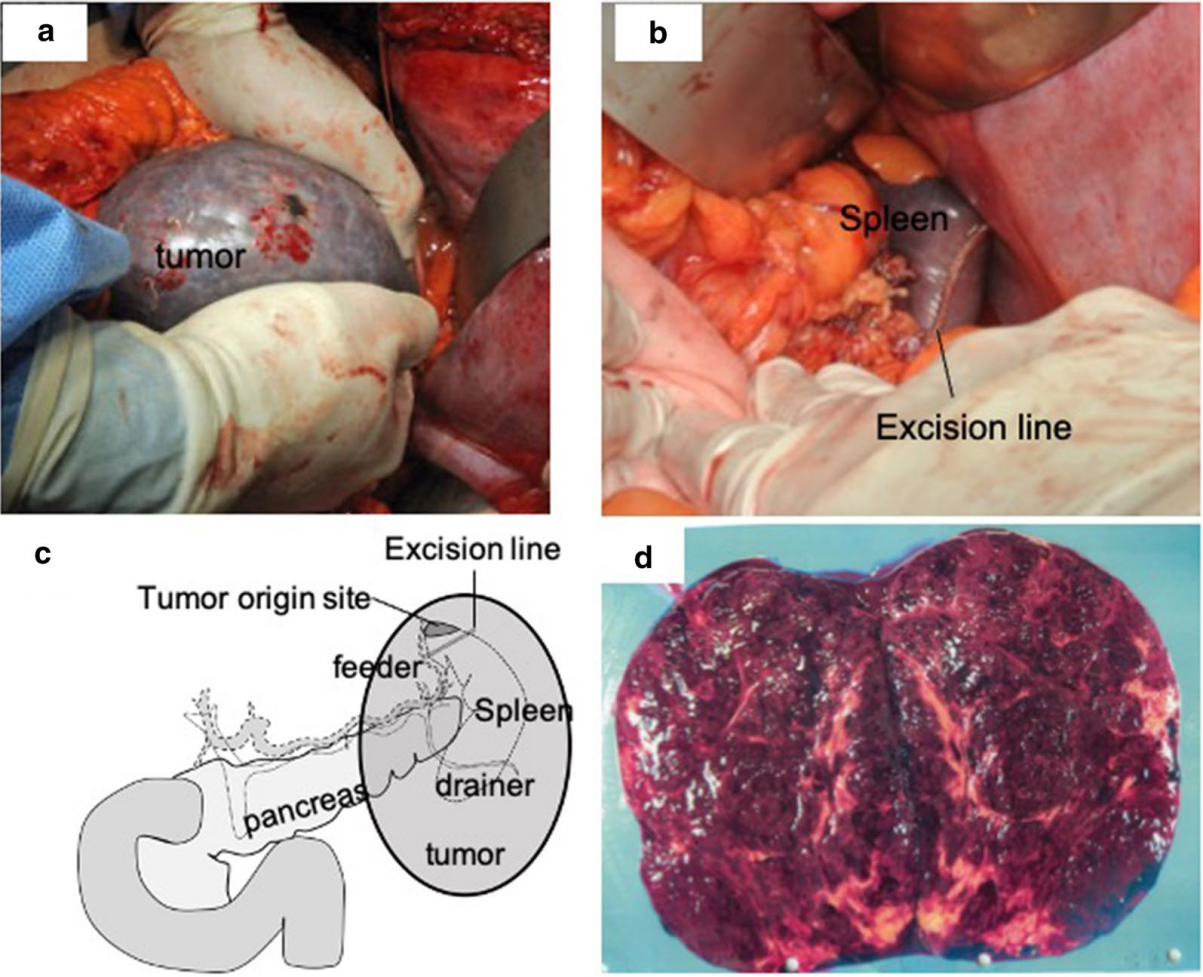
Operative findings and the excised specimen. **a** Operative findings revealed a smooth, soft tumor approximately 13 cm in the upper pole of the spleen. **b** Partial upper spleen resection using the ECHELON FLEX^®^ 60 powered stapler. **c** Schema showing tumor location, feeder, drainer vessel, and excision line. **d** The excised specimen was a smooth, extremely soft tumor of size 123 × 120 × 82 mm. The tumor remained in the splenic capsule, the cleaved surface was reddish brown, and a distressing yellow color was also observed

Partial resection of the spleen was performed. The abdomen was opened using an upper abdominal L-shaped incision. A giant tumor of approximately 13 cm with a smooth surface was observed in the upper left quadrant of the abdomen (Fig. 3a). The tumor was confirmed to be continuous with the upper spleen, and there was no invasion of the other organs. The vein draining the tumor encircling the left kidney was ligated and dissected. ECHELON FLEX^®^ 60 powered stapler (Ethicon Inc., Somerville, NJ, USA) was used to perform a partial resection of the spleen, including the tumor (Fig. 3b, c). Since we managed to preserve the splenic arteries and veins, no vascular treatment was performed at the splenic hilum. Before splitting the splenic parenchyma, it was pressed and thinned with the operator's finger for 5 min and then dissected using an automatic suturing device once. The operative time was 173 min. The volume of blood loss was 300 mL. The postoperative course was good, with no effect on the platelets, and the patient was discharged on the 7th postoperative day.

**Fig. 4:**
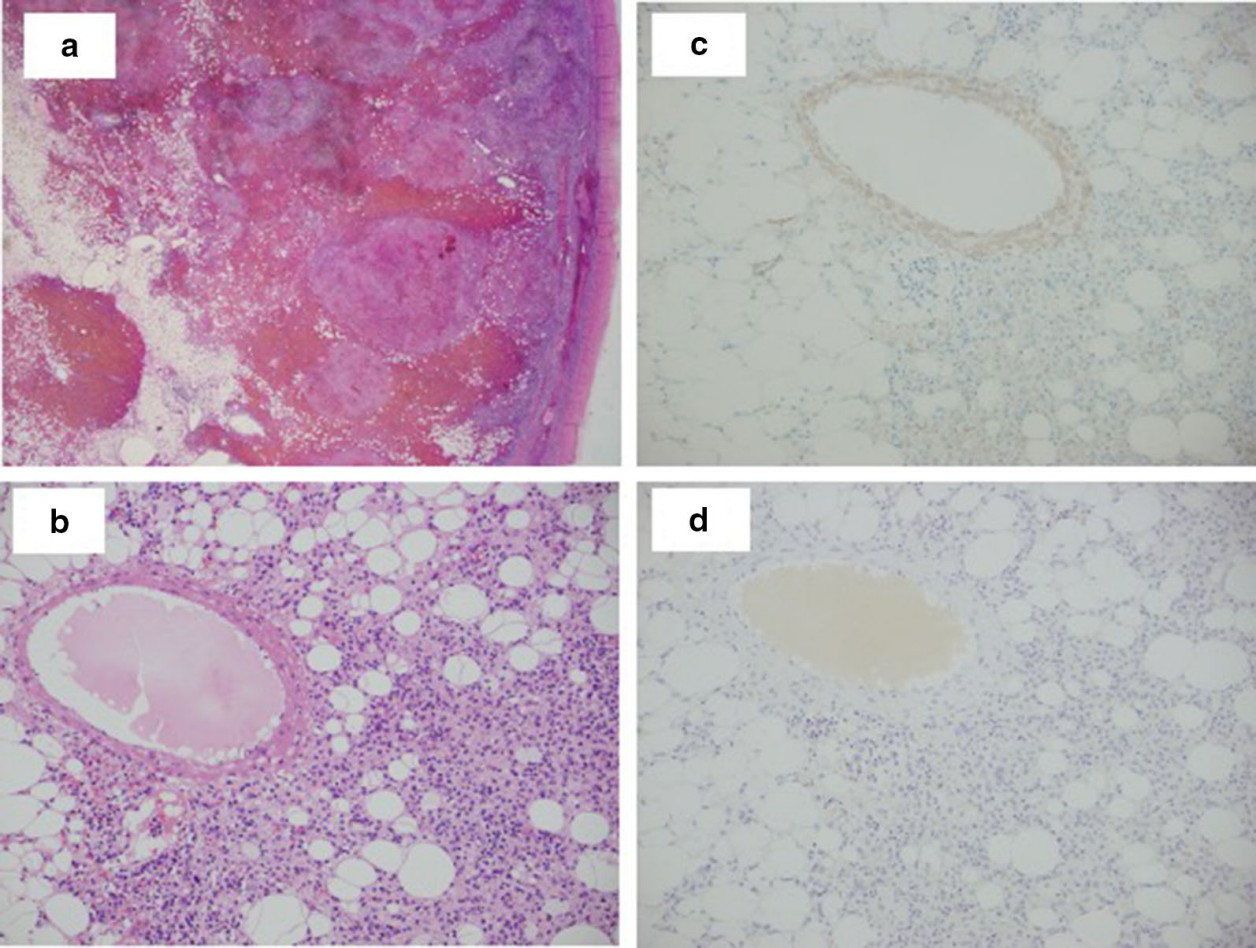
Pathological examination. **a**, **b** Hematoxylin and eosin stain: mature adipocyte proliferation and increased blood vessels of various sizes. **c** SMA: spindle-shaped cells were weakly positive and were smooth muscle cells. **d** HMB45: negative

The excised specimen was a smooth, extremely soft tumor measuring 123 × 120 × 82 mm. The cleaved surface of the tumor was reddish brown, and a distressing yellow color was observed (Fig. 3d). Pathological examination indicated mature adipocyte proliferation and increased number of blood vessels of various sizes. Additionally, spindle-shaped cell proliferation foci were found between the adipocytes and the surrounding blood vessels. Profuse leakage of erythrocytes from the blood vessels, hemosiderin deposition, and small round cell infiltration were also observed (Fig. 4a, b). Immunostaining exposed that the spindle-shaped cells were weakly positive for smooth muscle antibody (SMA), and were identified as smooth muscle cells (Fig. 4c). The adipocytes and spindle cells were negative for HMB 45, Melan A, MDM, and CDK4. However, some parts of the cells were positive for estrogen and progesterone receptors. Vascular endothelial cells were positive for CD31 and CD34 and negative for CD8. Based on the above-mentioned findings, the tumor was diagnosed as primary angiomyolipoma of the spleen.

Four years postoperatively, the patient has not faced any hematological problems, including those involving the platelets, and no new tumors have been identified in the spleen or other organs.

## Discussion

Angiomyolipoma is a mesenchymal benign tumor involving the smooth muscle tissue, mature adipocytes, as well as large and small blood vessels, the majority of which are present in the kidney [1]. Although angiomyolipoma that develops outside the kidney is rare, it has been reported to occur in various parts, such as liver, uterus, adrenal gland and retroperitoneum, among others [1–3]. However, we came across only one case report on angiomyolipoma originating from the spleen when searching in PubMed with the keywords “spleen” and “angiomyolipoma” [4]. This case concerned a 51-year-old man who had angiomyolipoma of the spleen and adenocarcinoma of the appendix, for which he underwent splenectomy and right hemicolectomy [4]. Splenic tumors can be either primary or metastatic, and primary tumors can be divided into the nonlymphatic and lymphatic categories. The most common nonlymphatic tumors are vascular tumors, including benign and malignant tumors. Angiomyolipoma is a nonlymphatic tumor. Metastatic tumors include melanoma, breast cancer, and lung cancer [5]. The average age of patients with retroperitoneal extrarenal angiomyolipomas is 45 years (range 22–80 years), with a male–female ratio of 1:5.3 [3]. Among the patients with renal angiomyolipomas, 30–40% may also exhibit features of TS. Similarly, 40–80% of the patients with TS are likely to develop renal angiomyolipoma [6–8]. However, the present case involved a 27-year-old man with no TS. Furthermore, a rare case of angiomyolipoma malignant transformation with lymph node metastasis has been reported [6–10]. However, all of these cases were associated with renal angiomyolipoma and TS. This case was not associated with malignant tumor, but Tang et al. reported an angiomyolipoma case of appendix carcinoid and Mogi et al. reported a case of gastric cancer [4, 11]. Although the number of cases of primary angiomyolipoma of the spleen is extremely small, it is necessary to consider the malignant complications.

CT is the most commonly used imaging technique in investigating angiomyolipomas [3]. As a typical imaging finding of angiomyolipoma, contrast-enhanced CT reveals a low-density part that reflects the fat component and a part that is strongly contrasted by enhancement. MRI shows a high signal in both T1- and T2-weighted images, and a decrease in the signal is observed in fat-suppressed images [3]. In this case, MRI could not be performed as the patient was claustrophobic, but CT exposed a low-concentration part reflecting the fat component and a solid part with a strong contrast effect. CT findings included differential diagnosis of the splenic angiomyolipoma, angioma including fat, and hamartoma. Angiomyolipoma is immunohistochemically positive for the melanocytic marker HMB45 and the smooth muscle marker SMA in many cases [12]. However, some cases are negative for HMB45. Tang et al. have reported a case that was positive for SMA but negative for HMB45 [4]. This case also consisted of three components, that is, smooth muscle, adipose tissue, and blood vessels. The patient was negative for HMB45 and positive for SMA; thus, splenic angiomyolipoma was diagnosed. These markers are useful for arriving at a definitive diagnosis, and preoperative biopsy may also aid in establishing the diagnosis. The protocol developed by Oesterling et al. is widely used to determine the treatment strategy for angiomyolipoma [6]. If the patient is asymptomatic, follow-up is sufficient. However, if the patient is symptomatic, treatment to alleviate the symptoms should be provided whenever the tumor size is ≥ 4 cm; if the tumor size is < 4 cm and the symptoms persist, treatment should be provided.

Treatment strategies for angiomyolipoma include surgery and arterial embolization, but the latter is less invasive and appears to be useful in preventing bleeding and achieving hemostasis [3]. The primary treatments for extrarenal retroperitoneal angiomyolipomas commonly involve surgery (surgical excisions, and less often, embolization of the tumor) [3]. Surgery should be considered when embolization is inadequate or when malignancy cannot be ruled out. The present case involved a giant tumor with a diameter of approximately 14 cm; there was a risk of rupture, and malignancy could not be ruled out.

Minja et al. have reported 16 cases of extrarenal MLs from 1982 to 2011 [3]. Nine cases had follow-up evaluations for 2–60 months after the surgical resection. All nine cases remained disease-free and asymptomatic at the last follow-up, and recurrence has not been documented. In retroperitoneal angiomyolipoma abutting the kidney, the previously reported recurrence was a case of metastasis to the liver and bone, which occurred 12 months after radical nephrectomy [13]. Metastatic masses were found on ultrasonography and CT. Extrarenal angiomyolipoma appears to have a good prognosis after surgical resection, but recurrence is possible in rare instances; hence, follow-up by imaging tests such as CT or ultrasonography may be necessary.

## Conclusions

We have reported the surgical treatment for a rare case of giant splenic angiomyolipoma in a young man. For spleen tumors, radical surgical excision or embolization of the lesion can be opted; nonetheless, if malignancy cannot be ruled out, surgical excision is necessary. We considered that partial splenic resection might be a feasible option for the treatment of a giant tumor as long as complete excision can be achieved. Extrarenal angiomyolipoma seems to have a favorable prognosis after surgical resection. Further evaluation, including diagnostic imaging and prognosis, is urgently needed to characterize primary angiomyolipoma of the spleen in a better manner. Moreover, follow-up will be required, including surveillance for malignancy.

## Data Availability

The authors declare that all data are available within the article.

## Abbreviations

AUS: Abdominal ultrasonography
CT: Computed tomography
MRI: Magnetic resonance imaging
